# Mesenchymal Stem Cell-Derived Exosomes Protect Muscle Loss by miR-145-5p Activity Targeting Activin A Receptors

**DOI:** 10.3390/cells10082169

**Published:** 2021-08-23

**Authors:** Kyung-Ah Cho, Da-Won Choi, Yu-Hee Kim, Jungwoo Kim, Kyung-Ha Ryu, So-Youn Woo

**Affiliations:** 1 Department of Microbiology, College of Medicine, Ewha Womans University, Seoul 07804, Korea; kyungahcho@ewha.ac.kr (K.-A.C.); akcmwns23@naver.com (D.-W.C.); rlawjddn1323@naver.com (J.K.); 2 System Biohealth BK21, Ewha Womans University, Seoul 07804, Korea; 3 Advanced Biomedical Research Institute, Ewha Womans University Seoul Hospital, Seoul 07804, Korea; kimyuhee@ewha.ac.kr; 4 Department of Pediatrics, College of Medicine, Ewha Womans University, Seoul 07804, Korea

**Keywords:** mesenchymal stem cell, exosomes, skeletal muscle, activin A

## Abstract

Skeletal muscle mass is decreased under a wide range of pathologic conditions. In particular, chemotherapy is well known for inducing muscle loss and atrophy. Previous studies using tonsil-derived mesenchymal stem cells (T-MSCs) or a T-MSC-conditioned medium showed effective recovery of total body weight in the chemotherapy-preconditioned bone marrow transplantation mouse model. This study investigated whether extracellular vesicles of T-MSCs, such as exosomes, are a key player in the recovery of body weight and skeletal muscle mass in chemotherapy-treated mice. T-MSC exosomes transplantation significantly decreased loss of total body weight and muscle mass in the busulfan-cyclophosphamide conditioning regimen in BALB/c recipient mice containing elevated serum activin A. Additionally, T-MSC exosomes rescued impaired C2C12 cell differentiation in the presence of activin A in vitro. We found that T-MSC exosomes possess abundant miR-145-5p, which targets activin A receptors, ACVR2A, and ACVR1B. Indeed, T-MSC exosomes rescue muscle atrophy both in vivo and in vitro via miR-145-5p dependent manner. These results suggest that T-MSC exosomes have therapeutic potential to maintain or improve skeletal muscle mass in various activin A elevated pathologic conditions.

## 1. Introduction

Despite significant progress in developing novel cancer treatments, chemotherapy is utilized for most tumors. Chemotherapeutic agents act primarily by antagonizing the essential mechanisms of cell division. These antiproliferative and cytotoxic drugs are highly effective in ablation of cancer cells, but are also responsible for dramatic toxicities in the host body [1]. It is commonly known that chemotherapy directly induces the loss of muscle mass and muscle strength in cancer patients. This condition, often referred to as cachexia, can persist for months to years following remission [2,3,4]. Cancer patients with low skeletal muscle mass during treatments have been reported to have a higher risk of mortality, increased cancer recurrence, and reduced quality of life [5,6]. Indeed, severe muscle loss after chemotherapy is an independent predictor of prognosis [7]. Therefore, strategies are required to limit chemotherapy-related toxicities such as muscle loss.

Activin A is a member of the transforming growth factor beta (TGF-β) family and a well-known negative regulator of skeletal muscle mass [8]. Activin A first binds to the type II activin receptors (ActR-IIA or ActR-IIB) on the cell surface, leading to the recruitment and phosphorylation of the type I activin receptor ActR-IB. Binding leads to subsequent signal transduction and causes upregulation of proteolytic genes [9,10]. Interestingly, elevated circulating activin A levels in cancer patients are related to cancer cachexia and reduced chemotherapy response [11]. Also, chemotherapeutic agents, such as busulfan, appear to be a direct inducer of activin A in vitro and in vivo [12]. Thus, targeting activin signaling may result in promising options to efficiently prevent skeletal muscle loss in pathological conditions such as chemotherapeutic cancer treatment.

We previously confirmed that human palatine tonsil-derived mesenchymal stem cells (T-MSCs) effectively recovered body weight in a chemotherapy-preconditioned bone marrow transplantation (BMT) mouse model [13,14]. A similar effect was observed when we used either T-MSCs or a conditioned medium from T-MSCs. Therefore, we hypothesized that extracellular vesicles such as exosomes may influence the recovery of body weight and skeletal muscle mass in chemotherapy. In this study, we investigated the effect of T-MSCs-derived exosomes on skeletal muscle in vitro and in vivo, particularly in an activin A abundant microenvironment such as chemotherapy treatment.

## 2. Materials and Methods

### 2.1. Animals

Female BALB/c mice at 8 weeks of age were purchased from OrientBio (Gyeonggi-do, Korea). All animals were maintained at temperatures of 21–23 °C with 51–54% humidity under pathogen-free conditions on a 12 h light/dark cycle with free access to food and water. All procedures were approved by the Ewha Womans University College of Medicine Animal Care and Use Committee (EUM 20-015).

### 2.2. Cell Culture

C2C12 mouse myoblasts were kindly provided by Dr. Song (Dongguk University, Gyeongju, Korea) and cultured in a growth medium consisting of Dulbecco’s Modified Eagle Medium (DMEM; Welgene, Daegu, Korea) supplemented with 20% fetal bovine serum (FBS; Welgene), 100 U/mL penicillin, and 100 μg/mL streptomycin at 37 °C in a humidified atmosphere of 5% CO_2_. The cells at 90% confluency were differentiated into myotubes by culturing in DMEM containing 2% horse serum (HS; Sigma-Aldrich, St. Louis, MO, USA), penicillin, and streptomycin for 5–7 days. To observe the effects of activin A or T-MSC exosomes on C2C12 cell differentiation, cells were treated with 50 ng/mL activin A on the first day of differentiation periods with or without various concentration of T-MSC exosomes (0.5, 1, 5, 10 μg/mL).

### 2.3. Isolation of Exosomes from T-MSCs

To isolate exosomes, a T-MSC-conditioned medium (T-CM) was generated. Established T-MSCs [14] were grown to 80–90% confluence in 100 mm tissue culture plates, cells were washed four times with phosphate-buffered saline (PBS), and the medium was replaced with serum-free DMEM. The medium was collected after 48 h of culture, centrifuged at 1300 rpm for 5 min, and passed through a 0.2-μm filter. The CM was concentrated to 20-fold of the original concentration by centrifugal filtration (cut-off of 3K, Amicon Ultra-15, Millipore, Bedford, MA, USA). Thereafter, 1/5 volume of ExoQuick-TC reagent (System Biosciences, Palo Alto, CA, USA) was added to T-CM and mixed by vigorous inverting. After incubation at 4 °C overnight, the mixture was centrifuged at 1500× *g* for 30 min at 4 °C. The supernatant was removed, and final centrifugation was performed for 5 min at room temperature. The visible exosome pellet was resuspended in PBS, quantified using the BCA protein assay kit (Thermo Fisher Scientific, Waltham, MA, USA), and stored at −80 °C.

### 2.4. Scanning Electron Microscopy (SEM)

Diluted exosomes were dropped on Poly L-lysine Cover glass and prefixed with 0.25% glutaraldehyde for 30 min. After several washes with PBS, samples were kept in 1% osmium tetroxide for 30 min for the final fixation. Subsequently, samples were washed and dehydrated through serial dilutions with ethanol and critical point drying (CPD). Finally, the samples were mounted onto stubs, sputter-coated with gold by Quorum (Quorum Technologies, East Sussex, UK) and examined with SEM Sigma-300 (ZEISS, Oberkochen, Germany).

### 2.5. Transmission Electron Microscopy (TEM)

Purified exosomes were diluted to 1:1000 in PBS. Five microliters of diluted exosomes were dropped on Formvar-carbon coated electron microscope (EM) grids. The grids were stained with 2% uranyl acetate and were removed using filter paper. Finally, the grids were viewed using a H-7650 TEM (Hitachi, Tokyo, Japan) at a voltage of 80 kV. Digital images with scale bar provided were captured at a magnification of 70,000–200,000×.

### 2.6. Immunoblotting of Exosome Markers

Immunoblots were performed on T-MSC exosomes and T-MSC lysates to check the expression of exosome markers CD63 and CD81. Five micrograms of exosomes and T-MSC proteins were loaded per lane, and the blotted membranes were incubated overnight with a primary antibody against CD63 (ab193349, Abcam, Cambridge, UK) and CD81 (sc-166029, Santa Cruz Biotechnology, Santa Cruz, CA, USA). After intensive washing, the membranes were incubated with the secondary antibody (anti-mouse IgG, Sigma Aldrich). The images were developed using SuperSignal West Femto Substrate (Thermo Fisher Scientific) and scanned using ImageQuant LAS 3000 (GE Healthcare, Little Chalfont, UK).

### 2.7. Quantitative Reverse Transcription-Polymerase Chain Reaction (qRT-PCR)

For microRNA (miRNA), exosomal RNAs were isolated from T-CM using Exo2D for RNA assay kit (Exosome Plus, Gyeonggi-do, Korea) according to manufacturer’s instruction. Next, miRNAs were polyadenylated and subsequently converted into complementary DNA (cDNA) using MystiCq MicroRNA cDNA Synthesis Mix kit (Sigma-Aldrich) in accordance with manufacturer’s instruction. Real-time PCR analysis was performed on a StepOnePlus instrument (Applied Biosystems, Foster City, CA, USA) using a SensiFAST SYBR Hi-ROX kit (Bioline, London, UK). MicroRNA 145-5p expression was normalized to the control miRNA, RNU6-1. The primers for miR-145-5p and RNU6-1 were purchased from Sigma-Aldrich.

For detection of muscle relevant genes, experimental mice quadriceps, C2C12 cells and differentiated myotubes were collected, followed by extraction of RNA using TRIzol (Thermo Fisher Scientific). Complementary DNA was synthesized using reverse transcription reagent (ELPIS-Biotech, Daejeon, Korea) according to the manufacturer’s instructions. Real-time PCR analysis was performed as previously described. All gene expression values (MyoD, Myh1, ACVR2A, ACVR1B, MuRF1, and Atrogin1) were normalized to the GAPDH reference gene. All qRT-PCR primers used are listed in Table 1. For ACVR2A and ACVR1B, the amplicon was also produced by end-point RT-PCR using same primers.

### 2.8. Prediction of Target Genes

The miRNA target gene prediction was performed by TargetScanHuman 7.2 (http://www.targetscan.org/vert_72/, accessed on 4 January 2021).

### 2.9. MTT Assay

To check viability of C2C12 cells in the presence of T-MSC exosomes, 3-(4,5-dimethylthizol-2-yl) 2,5-diphenyl tetrazolium bromide (MTT, Sigma-Aldrich) assay was performed. C2C12 cells were plated onto 96 well microtiter plates and treated with T-MSC exosomes at a various concentration (0.5, 1, 5, 10, and 20 μg/mL). After 24 h, MTT and dimethyl sulfoxide were sequentially added to all the wells to dissolve the dark blue formazan crystals.

### 2.10. Transfection

To inhibit the activity of T-MSC exosome-derived miR-145-5p during myotube differentiation, C2C12 cells were transfected with a miR-145-5p specific inhibitor. When C2C12 cells reached 90% confluency, culture media was replaced with differentiation media. Cells were transfected with miR-145-5p specific inhibitor oligonucleotides (Bioneer, Daejeon, Korea) using Metafectene reagent (Thermo Fisher Scientific) in accordance with the manufacturer’s instructions. Non-targeted control oligonucleotides (Bioneer) were used as negative controls. After 5 h, the cells were treated with T-MSC exosomes (5 μg/mL) and differentiated into myotubes for 5 days in the presence of activin A (50 ng/mL).

### 2.11. Immunofluorescence Staining

C2C12 cells differentiated under various conditions were subjected to immunofluorescence staining. Cells were differentiated with or without activin A (50 ng/mL) for 5–7 days. Furthermore, cells transfected with micro RNA inhibitors were differentiated in the presence of activin A and T-MSC exosomes (5 μg/mL). All cells were cultured in a Lab-Tek Chamber Slide (Thermo Fisher Scientific). After washing with PBS, cells were fixed with 4% formaldehyde for 15 min and then incubated with primary antibodies against myogenin (Abcam, Cambridge, MA, USA) overnight at 4 °C. After that, washed slides were incubated with DyLight-conjugated anti-mouse IgG H&L (Abcam) for 1 h at room temperature in the dark. After washes, slides were mounted with DAPI solution (Vector Laboratories, Burlingame, CA, USA) and examined using a fluorescent microscope (Olympus, Tokyo, Japan).

### 2.12. Chemotherapy in Mice

Female BALB/c recipient mice received busulfan (Bu, 20 mg/kg/day) daily for 4 days, followed by cyclophosphamide (Cy, 100 mg/kg/day) daily for 2 days via intraperitoneal injection. On day 3 during chemotherapy, mice were injected with T-MSC exosomes (30 μg/mouse) via lateral tail vein. To block activity of exosomes-derived mi-R-145-5p, miR-145-5p specific inhibitor (Bioneer) or non-targeting control inhibitor (Bioneer) were formulated with an in vivo jetPEI transfection reagent (Polyplus Transfection, Illkirch, France), and 100 μg of oligonucleotides were administered by intraperitoneal injection. Mice were sacrificed 4–5 days after the last cyclophosphamide injection. All mice were examined daily for the experimental period and weight loss was recorded. At the end of the experimental periods, mice were sacrificed and quadricep masses were measured and processed for further evaluation including histology and gene expression.

### 2.13. Histology

Specimens from experimental mice quadriceps were fixed with 4% formaldehyde and embedded in paraffin. Sections (5 μm thickness) were mounted on slides and stained with hematoxylin and eosin (H&E) for histological evaluation.

### 2.14. ELISA

To detect circulating activin A, serum was collected from normal BALB/c mice, Bu-Cy treated mice, and exosome injected Bu-Cy mice at the end of the experimental period. The levels of activin A were determined using the Human/Rat/Mouse Activin A immunoassay kit (R&D Systems, Minneapolis, MN, USA) according to the manufacturer’s instructions.

### 2.15. Statistics

Data are presented as mean ± standard error of the mean (SEM). Statistical significance was analyzed by one-way ANOVA or Student’s *t*-test using GraphPad PRISM 7 software (GraphPad Software Inc., San Diego, CA, USA). For all analyses, a *p* < 0.05 was considered as statistically significant.

## 3. Results

### 3.1. T-MSC Exosomes Recovered Weight and Muscle Loss in Bu-Cy Treated Mice

**Figure 1:**
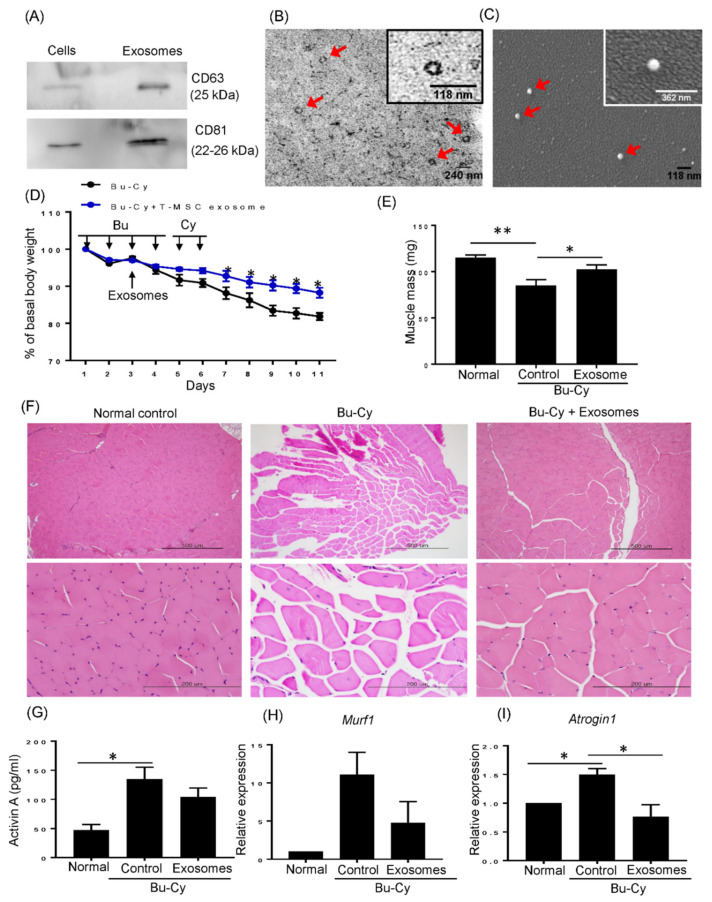
T-MSC exosomes alleviate loss of total body weight and skeletal muscle mass in Bu-Cy treated mice. (**A**) Immunoblotting was performed with T-MSC lysates and T-MSC exosomes. The expression of CD63 and CD81 were detected. (**B**) Exosomes isolated from the T-MSC-conditioned medium were visualized via transmission electron microscopy. 100,000× original magnification and 240 nm size bar. (**C**) Exosomes isolated from the T-MSC-conditioned medium were visualized via scanning electron microscopy. 70,000× original magnification and 118 nm size bar. Inserted image is enlarged exosome. The nanoparticles were between 40–100 nm and are indicated by arrows. (**D**) The total body weight of experimental BALB/C recipients was monitored over the study duration. (**E**) The quadriceps weight of each experimental mouse was measured at the end of the study period. The data are presented as the mean ± SEM (* *p* < 0.05, ** *p* < 0.01). (**F**) Images of H&E-stained sections of quadriceps from experimental mice (100× magnification in upper panel; 400× magnification in lower panel). (**G**) Serum activin A levels were assessed in control, Bu-Cy, T-MSC exosome injected Bu-Cy mice by ELISA. The data are presented as the mean ± SEM (* *p* < 0.05). The mRNA expression of MuRF1 (**H**) and Atrogin1 (**I**) on quadriceps from each experimental mouse was detected by qRT-PCR. The data are presented as the mean ± SEM (* *p* < 0.05).

Previously, we observed T-MSCs and T-CM recovered body weight in a chemotherapy-preconditioned BMT mouse model. To investigate whether MSC-secreted exosomes can act on such T-MSC-mediated weight recovery, T-CM was concentrated for further isolation of exosomes. First, the exosomes were characterized by CD63 and CD81 expression (Figure 1A).

The diameter of exosomes was confirmed to be less than 100 nm by TEM (Figure 1B) and SEM analysis (Figure 1C). To identify the effects of T-MSC exosomes, mice were treated with Bu-Cy for cytotoxic conditioning as previously established and injected with T-MSC exosomes on the third day during Bu-Cy treatment. Rapid weight loss was observed until 4 days post-Bu-Cy injection, but T-MSC exosome injected Bu-Cy mice showed significantly milder weight loss (Figure 1D). This weight loss was accompanied with skeletal muscle loss as indicated by Figure 1E. The quadriceps muscle mass appeared to be decreased by Bu-Cy treatment, and T-MSC exosomes significantly rescued the chemotherapy induced muscle loss. Furthermore, quadriceps muscle tissue exhibited a clear disruption of muscle fibers and increased internalized nuclei in Bu-Cy mice, but T-MSC exosomes reversed muscle disruption induced by the conditioning regimen (Figure 1F). As activin A is a negative regulator of muscle mass, and elevated levels of activin A are reported during chemotherapy, we confirmed the levels of activin A in serum of each experimental mouse. As shown in Figure 1G, Bu-Cy treatment significantly increased circulating activin A. Thus, we detected expression of MuRF1 and Atrogin1, which are known activin A-induced muscle atrophy genes [15,16]. The expression of MuRF1 was upregulated in Bu-Cy treatment and decreased by T-MSC exosomes. Additionally, T-MSC exosomes significantly reduced Atrogin1 expression under Bu-Cy treatment (Figure 1H,I).

### 3.2. Activin A Disturbs Myotube Differentiation

**Figure 2:**
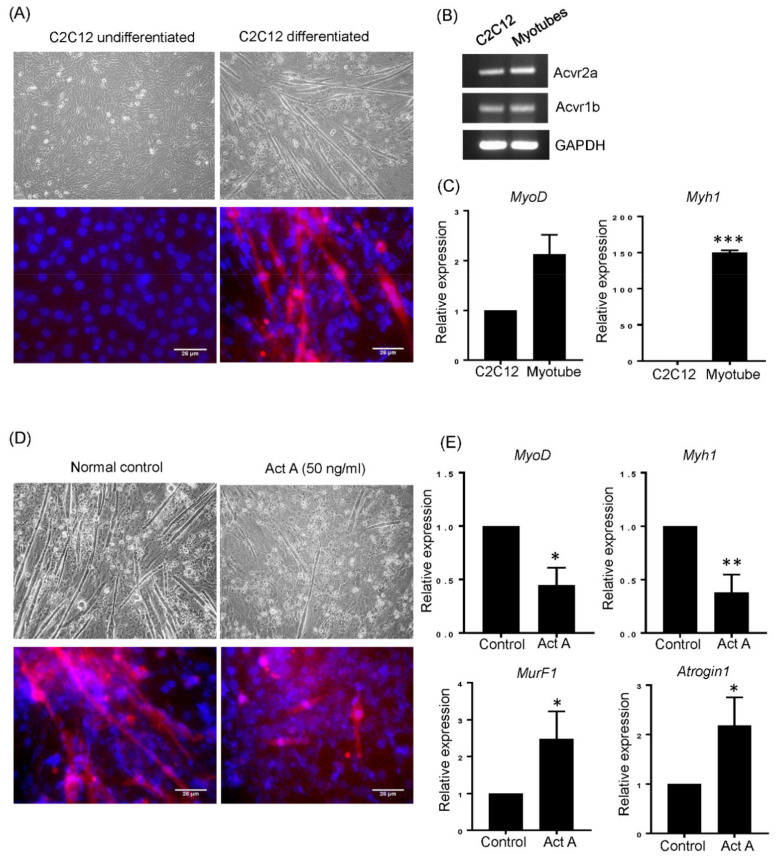
Activin A treated C2C12 cells showed impaired differentiation capacity. (**A**) C2C12 myoblasts (left) were differentiated and formed myotubes (right). The morphological changes were observed by phase contrast microscopy (Original magnification, 200×, upper panel). The expression of myogenin was confirmed by immunofluorescence staining (Myogenin (red) and DAPI (blue), lower panel). (**B**) C2C12 myoblasts and myotubes were harvested and the expression of activin A receptors ACVR2A and ACVR1B were detected by RT-PCR. (**C**) The expression of myogenic markers MyoD and MYH1 were detected by qRT-PCR. The data are presented as the mean ± SEM (*** *p* < 0.001). (**D**) C2C12 myoblasts were differentiated in the presence or absence of activin A (50 ng/mL) for 5 days, and visualized by phase contrast microscopy (Original magnification, 200×, upper panel). The expression of myogenin was confirmed by immunofluorescence staining (Myogenin (red) and DAPI (blue), lower panel). (**E**) The expression of myogenic makers (MyoD and MYH1) and atrophy genes (MuRF1 and Atrogin1) was detected by qRT-PCR in differentiated C2C12 cells without activin A (control) and in C2C12 cells differentiated in the presence of activin A. The data are presented as the mean ± SEM (* *p* < 0.05, ** *p* < 0.01).

We speculated that activin A may directly affect myotube differentiation. Therefore, we investigated the effect of activin A on differentiation capacity of C2C12 cells into myotubes, as well as the expression of activin A responsive muscle atrophy genes, MuRF1 and Atrogin1. C2C12 myoblasts started to form myotubes on days 2–3 under differentiation conditions and were fully differentiated by day 7 with expression of myogenin protein, a skeletal muscle marker (Figure 2A). Both C2C12 myoblasts and differentiated myotubes constitutively expressed activin A receptors, ACVR2A and ACVR1B (Figure 2B). Differentiated myotubes also highly expressed muscle-specific transcription factor, MyoD, and mature skeletal muscle marker, *MYH1* (Figure 2C). When 50 ng/mL of activin A was added during differentiation periods, muscle fiber formation and myogenin expressing tubes were decreased as compared to non-treated control C2C12 cells (Figure 2D). This impairment of fiber formation was accompanied with significantly lower expression of *MyoD* and MYH1 compared to control C2C12 cells. Furthermore, the expression of *MuRF1* and *Atrogin1* were upregulated by activin A treatment, as shown in Figure 2E.

### 3.3. T-MSC Exosomes Highly Express Activin A Receptor Targeting has-miR-145-5p

**Figure 3:**
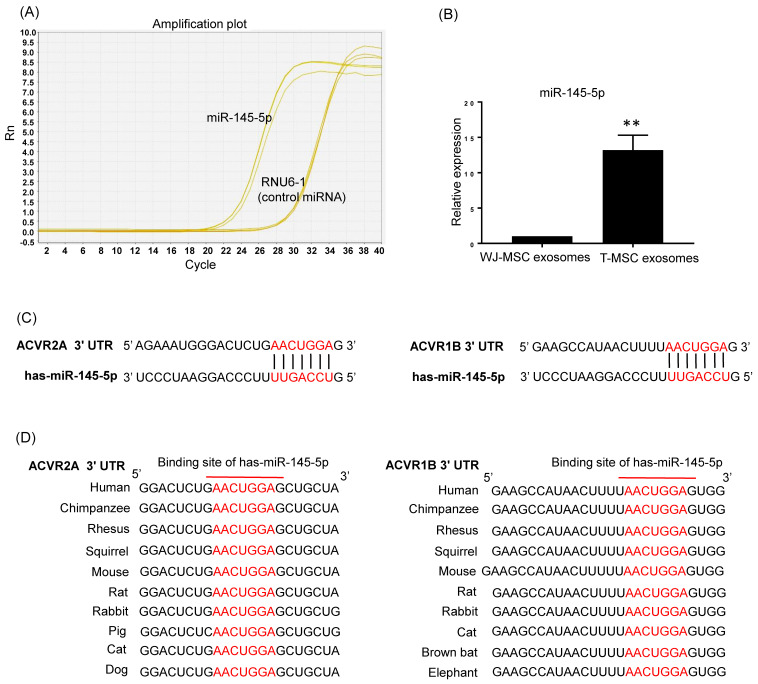
has-miR-145-5p is highly expressed in T-MSCs exosomes and directly targets ACVR2A and ACVR1B. (**A**) Amplification plot of has-miR-145-5p and control miRNA, RNU6-1 in T-MSCs exosomes from qRT-PCR (**B**) The relative expression of has-miR-145-5p in Wharton’s jelly MSCs exosomes and T-MSCs exosomes was quantified by qRT-PCR. The data are presented as the mean ± SEM (** *p* < 0.01). (**C**) The predicted binding site of has-miR-145-5p in ACVR2A 3′-UTR and ACVR1B 3′-UTR. (**D**) Sequence alignment of the mature has-miR-145-5p binding sites in multiple species.

Because T-MSC exosomes efficiently recovered muscle loss and relevant gene expression in vivo, we hypothesized that components of exosomes, such as miRNA, possibly act on muscle mass regulation. Recently we found unique profiles of T-MSC exosomes including a highly expressed exosomal miRNA, has-miR-145-5p. T-MSC exosomes showed constitutive expression of has-miR-145-5p and the expression was significantly higher compared to Wharton’s jelly MSCs exosomes (Figure 3A,B). To further explore the possible molecular mechanism underlying muscle mass regulation, we predicted putative miR-145-5p target sequences using the TargetScanHuman 7.2 database. The seed sequence of has-miR-145-5p perfectly matches the 3′ untranslated region (UTR) of ACVR2A and ACVR1B, suggesting that activin A receptors are potential targets of has-miR-145-5p (Figure 3C). Furthermore, we found that the potential binding site of has-miR-145-5p was highly conserved among mammalian ACVR2A and ACVR1B genes (Figure 3D). These results indicate that has-miR-145-5p in T-MSC exosomes may affect activin A receptor-mediated response in muscle.

### 3.4. T-MSC Exosomes Restored Muscular Differentiation Impairment Induced by Activin A

**Figure 4:**
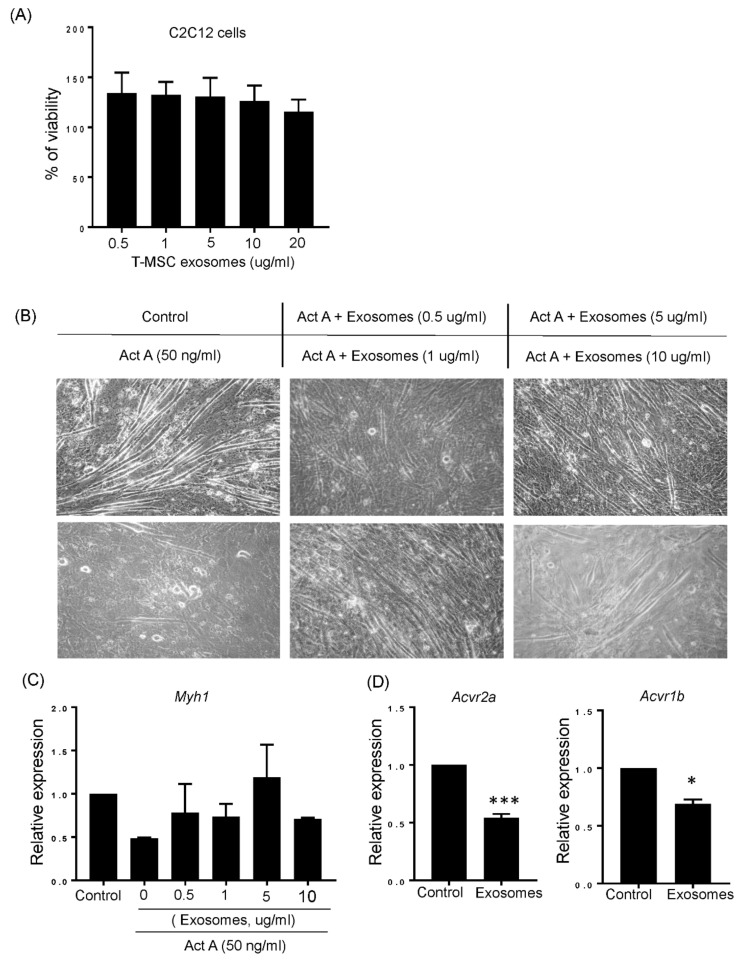
T-MSC exosomes recovered activin A induced impairment of C2C12 cells differentiation. (**A**) Viability of C2C12 cells cultured in the presence of T-MSC exosomes at the indicated concentrations for 24 h was measured by MTT assay. (**B**) C2C12 cells were differentiated into myotubes in the presence or absence of activin A. T-MSC exosomes at various concentrations were also added for differentiation periods. The phase contrast microscopy image was taken on day 5 of differentiation (Original magnification, 200×). (**C**) The expression of MYH1 was detected in differentiated C2C12 cells for 5 days under indicated conditions by qRT-PCR. (**D**) The expression of ACVR2A and ACVR1B in C2C12 cells treated with T-MSC exosomes (5 μg/mL) was detected by qRT-PCR. The data are presented as the mean ± SEM (* *p* < 0.05, *** *p* < 0.001).

The maintenance of muscle mass in T-MSC exosomes in vivo led to us to confirm the effect of T-MSC exosomes on muscular differentiation in C2C12 myoblasts. Prior to treating exosomes, potential toxicity was checked by measuring cellular viability. As shown in Figure 4A, C2C12 cells treated with various concentrations of T-MSC exosomes had increased cell viability over 100%, as compared to untreated C2C12 cells set to 100%. Next, we tested whether T-MSC exosomes improved muscular differentiation in the presence of activin A. Confluent C2C12 myoblasts started differentiation and were treated with 50 ng/mL activin A and various concentrations of T-MSC exosomes (0.5, 1, 5, 10 μg/mL) on the first day of differentiation. After 5 days, cells treated with activin A alone showed limited formation of myotubes. Conversely, cells exposed to T-MSC exosomes formed abundant myotubes even in the presence of activin A (Figure 4B). The expression of the MYH1 skeletal muscle marker was also recovered in C2C12 cells treated with T-MSC exosomes under activin A exposure. In particular, we found that T-MSC exosomes most effectively increased MYH1 expression at a concentration of 5 μg/mL (Figure 4C). Considering the abundance of has-miR-145-5p in T-MSC exosome and potential activity on activin A receptors, ACVR2A and ACVR1B, we confirmed the expression of ACVR2A and ACVR1B in C2C12 cells treated with T-MSC exosomes for 24 h. Figure 4D shows that ACVR2A and ACVR1B were significantly downregulated in C2C12 cells with T-MSC exosomes added. Thus, T-MSC exosomes may improve activin A-induced impaired muscular differentiation, possibly by regulating activin A receptor expression.

### 3.5. T-MSC Exosomes Regulate Muscular Response to Activin A via has-miR-145-5p Activity

**Figure 5:**
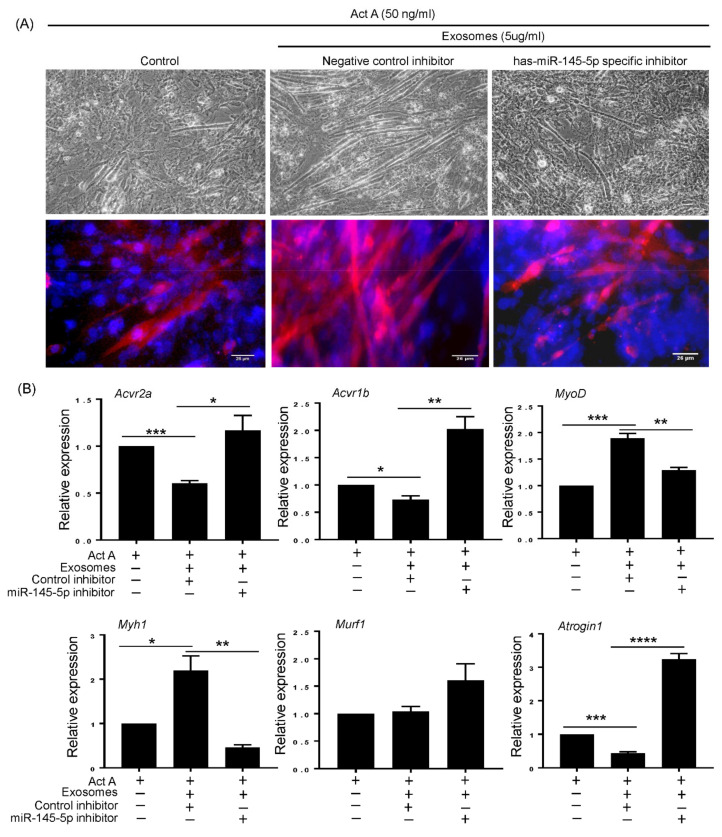
The supportive effect of T-MSC exosomes on muscular differentiation is reduced by has-miR-145-5p inhibitor. (**A**) C2C12 cells were differentiated into myotubes in the presence of activin A and T-MSC exosomes. To observe the effect of has-miR-145-5p, cells were transfected with non-targeted control inhibitor or has-miR-145-5p specific inhibitor. The phase contrast microscopy image was taken on day 5 of differentiation (Original magnification, 200×, upper panel). The expression of myogenin was confirmed by immunofluorescence staining (Myogenin (red) and DAPI (blue), lower panel). (**B**) The expression of activin A receptors (ACVR2A and ACVR1B), myogenic markers (MyoD and MYH1), and atrophy genes (MuRF1 and Atrogin1) were detected in experimental C2C12 cells by qRT-PCR. The data are presented as the mean ± SEM (* *p* < 0.05, ** *p* < 0.01, *** *p* < 0.001, **** *p* < 0.0001).

To clarify the molecular mechanism underlying T-MSC exosome-mediated muscle regulation, we investigated whether has-miR-145-5p was a key player in T-MSC exosomes supporting muscular differentiation. Supplementation of T-MSC exosomes with a non-targeted miRNA inhibitor promoted myotube generation in activin A-treated C2C12 cells. Alternatively, treatment with a has-miR-145-5p-specific inhibitor abrogated the supportive effect of T-MSC exosomes on myotube formation (Figure 5A, upper panel). These myotube formation was also confirmed via expression of myogenin protein (Figure 5A, lower panel). When we investigated gene expression of each experimental cell, changes in myogenic markers, activin A responsive atrophy genes, and activin A receptors were observed (Figure 5B). The expression of MyoD and MYH1 was increased by treatment with T-MSC exosomes and a non-targeted miRNA inhibitor, but treatment with a has-miR-145-5p-specific inhibitor significantly reversed T-MSC exosomes-induced gene expression. Although MuRF1 atrophy gene expression was not affected by T-MSC exosomes alone, T-MSCs exosomes supplemented with has-miR-145-5p-specific inhibitor upregulated MuRF1 expression. Additionally, Atrogin1 expression was downregulated by T-MSC exosomes in a has-miR-145-5p-dependent manner. These results correlate with the decreased expression of activin A receptors, ACVR2A and ACVR1B, by T-MSC exosomes via has-miR-145-5p activity.

### 3.6. T-MSC Exosomes Rescue Body Weight Loss and Skeletal Muscle Loss via has-miR-145-5p Activity In Vivo

**Figure 6:**
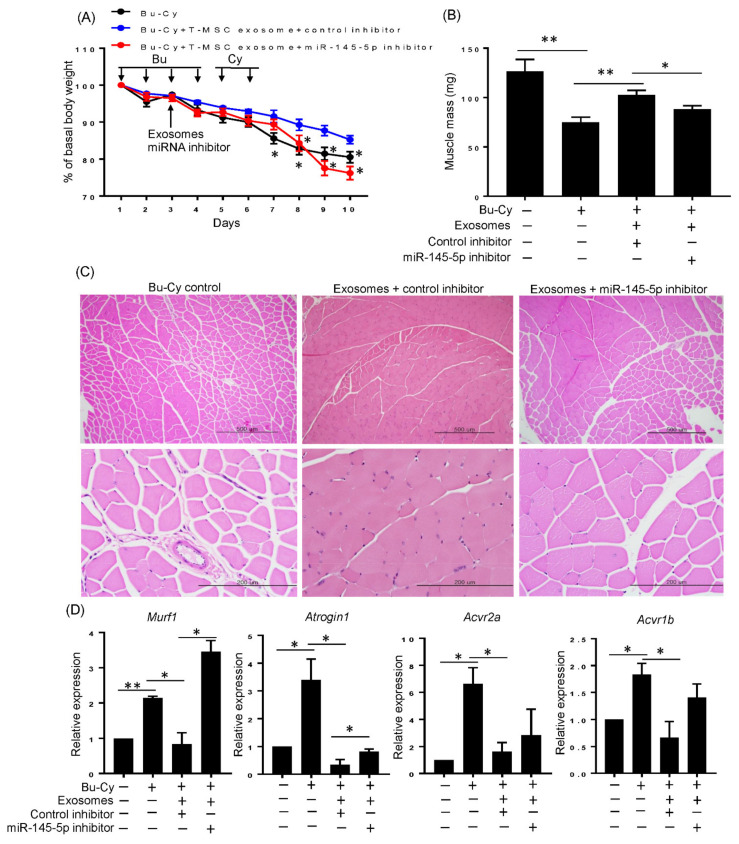
T-MSC exosomes alleviate loss of total body weight and skeletal muscle mass in Bu-Cy treated mice via has-miR-145-5p dependent manner. (**A**) The total body weight of experimental BALB/C recipients was monitored over the study duration. (**B**) The quadriceps weight of each experimental mouse was measured at the end of the study period. (**C**) The quadriceps from each experimental mouse was stained with H&E for histologic observations (100× magnification in upper panel; 400× magnification in lower panel). (**D**) The expression of MuRF1, Atrogin1, ACVR2A, and ACVR1B on quadriceps from each experimental mouse was detected by qRT-PCR. The data are presented as the mean ± SEM (* *p* < 0.05, ** *p* < 0.01).

The in vitro experimental results prompted us to investigate whether T-MSC exosomes attenuate in vivo skeletal muscle loss in a has-miR-145-5p-dependent manner. We injected T-MSC exosomes supplemented with nonspecific or miR-145-5p-specific inhibitors during Bu-Cy treatment. Mice injected with T-MSC exosomes and nonspecific inhibitor on the third day showed significantly inhibited weight loss starting the day after their last Cy treatment. However, these effects were diminished in mice that received T-MSC exosomes and miR-145-5p inhibitor starting two days after their last Cy treatment. The co-administration of T-MSC exosomes and miR-145-5p inhibitor resulted in a sharp decrease in weight towards the end of the experiment (Figure 6A). T-MSC exosomes in combination with the has-miR-145-5p inhibitor also blunted the muscle mass of each experimental mouse, as shown in Figure 6B. Furthermore, T-MSC exosome injected mice had milder quadriceps muscle fiber disruption induced by Bu-Cy when activity of miR-145-5p was normally maintained (Figure 6C). Lastly, co-administration with the has-miR-145-5p-specific inhibitor and T-MSCs exosomes in Bu-Cy-treated mice reversed the expression of MuRF1 and Atrogin1 atrophy genes (Figure 6D). Mice treated with Bu-Cy showed significant increases in activin A receptors, ACVR2A, and ACVR1B, indicating that the state of the tumor microenvironment (such as activin A increase under chemotherapy) could augment activin A responsiveness. Parallel with in vitro results, T-MSC exosomes significantly downregulated ACVR2A and ACVR1B in Bu-Cy mice, possibly explaining how atrophy genes are attenuated by T-MSC exosomes. Also, the decrease of activin A receptors by T-MSC exosomes were milder when the has-miR-145-5p inhibitor was co-administered (Figure 6D).

## 4. Discussion

In this study, we demonstrated that T-MSC exosomes alleviated loss of muscle mass in cytotoxic chemotherapeutic conditions by modulating activin A signaling. Bu-Cy treated mice underwent continuous weight loss and muscle wasting that was accompanied by an increase of atrophy genes expression in skeletal muscle tissue. We observed that circulating activin A, a well-known negative regulator of skeletal muscle mass, was increased in Bu-Cy mice. Also, activin A treatment inhibited muscular differentiation of C2C12 myoblasts in vitro. Importantly, we showed that T-MSC exosomes contain abundant has-miR-145-5p that targets activin A receptors, ACVR2A and ACVR1B. Indeed, T-MSC exosomes reversed activin A-induced differentiation reduction in C2C12 myoblasts by targeting ACVR2A and ACVR1B in a has-miR-145-5p-dependent manner. Lastly, Bu-Cy mice injected with T-MSC exosomes showed recovery of total body weight and muscle mass via has-miR-145-5p activity. Thus, T-MSC exosomes could support maintaining skeletal muscle mass within an activin A abundant cytotoxic microenvironment.

Skeletal muscle mass is dependent on diverse conditions, including aging, disuse, cachexia, denervation, and burns. Increased protein degradation due to reduced activity, defective neural input, or increased proteolysis in pathologic condition cause muscle atrophy [16]. Growing evidence suggests that signaling by activin A, a negative regulator of skeletal muscle mass, is upregulated in catabolic diseases and contributes to muscle wasting [17,18]. A mouse genetic study originally indicated the correlation of high circulating levels of activin A and severe muscle wasting. The biological activity of activin A is negatively regulated by inhibin A, and genetic inactivation of *Inha* resulted in elevated levels of activin A. Increased activin A caused a severe cachexia phenotype, including muscle wasting and decreased survival [19]. Subsequent studies have confirmed that elevated serum levels of activin A promote muscle atrophy and cachexia in mice [20,21]. An important role for activin A has also been established in human cancer cachexia [22]. Additionally, other reports have indicated that serum concentrations of activin A increase with age [23,24].

Activin A first binds to the type II activin receptors (ACVR2A or ACVR2B) on the cell surface, leading to the recruitment and phosphorylation of the type I activin receptor ACVR1B. Thus, both type II and type I receptors are equally required for full activation of activin A signaling. Binding to these receptors activates downstream processes resulting in Smad2/3 and FoxO1 nuclear translocation and transcriptional upregulation of Atrogin1 and MuRF1genes which code proteolytic enzymes [10,15,16]. Atrogin1 and MuRF1 are E3 ubiquitin ligases expressed in skeletal muscle that polyubiquitinate proteins target for proteolysis by the 26S proteasomes [25,26]. There are correlations between the onset of muscle atrophy and the increase in Atrogin1 and MuRF1 [27].

While current life expectancies have been extended, the incidence of intractable diseases is also increasing. Skeletal muscle mass is critically affected by such pathologic conditions including the disease itself or disease treatments that ultimately induce atrophy. Maintaining muscle mass throughout one’s lifespan could improve overall quality of life, increase muscle functionality, and potentially overcome disease. Thus, efficient therapeutics to restore or maintain skeletal muscle mass across various conditions are needed.

MSCs are a promising therapeutic option for a wide range of tissue engineering and regenerative medicine applications via their regenerative secretomes and differentiation capacity toward multiple lineages. Regarding muscle regenerations, T-MSCs showed promising evidence in several studies. They were differentiated into myogenic cells and effectively regenerated skeletal muscle following injury in vivo [28]. Moreover, T-MSC transplantation has also been studied in the Tr-J mouse model for Charcot–Marie–Tooth disease type 1A (CMT1A). Transplantation of T-MSC-derived myocytes into the gastrocnemius muscle in Tr-J mice enhanced motor function and recovered action potential amplitudes in muscle [29]. These results demonstrate that T-MSCs have the potential to regenerate skeletal muscle tissues in muscle-affected pathologic conditions.

Previously, we observed that co-transplantation with T-MSCs rapidly recovered total body weight in the chemotherapy-preconditioned BMT mouse model. The parallel effects of transplantation of T-MSCs and T-CM strongly indicate that secreted extracellular vesicles, such as exosomes, may improve weight loss-induced by Bu-Cy chemotherapy. Although numerous experimental and clinical studies demonstrated beneficial effects of MSCs as regenerative therapeutics, there are some shortcomings. Unwanted differentiation of transplanted MSCs and potential malignant transformation have been identified as the most important safety issues [30]. Also, MSCs express major histocompatibility complex (MHC) class I molecules. In MHC-class I-mismatched recipients MSCs may elicit strong allogeneic immune responses that could result in life-threatening aggravation of on-going inflammation [31]. Therefore, expanding the utility of MSCs, including cell free therapy using exosomes, could overcome the potential limitations in MSCs therapy. Currently, it is believed that MSCs achieve a therapeutic effect in vivo mainly through paracrine signaling. Exosomes are the key bioactive vesicles responsible for the paracrine effects of MSCs. Exosomes regulate many physiological and pathological processes by affecting the survival, proliferation, migration, and gene expression of recipient cells, and by reprograming targeted cell behaviors [32]. Exosomes contain a cargo of genetic materials (mRNA, miRNA, pre-miRNA, and other noncoding RNA) and proteins that are transferred to and released into target cells [33,34]. Our reproducible effects of T-MSC exosomes on weight recovery in Bu-Cy mice proved that exosomes are critical components of T-MSCs activity. We noticed that miR-145-5p is abundant in T-MSC exosomes, and specifically target activin A receptors. The highly preserved binding sequence of miR-145-5p against ACVR2A and ACVR1B in multiple species support the effectiveness of miR-145-5p on activin A mediated cellular response. Indeed, our in vivo and in vitro results indicate T-MSC exosomes affect activin A responsive gene expression of MuRF1 and Atrogin1 by targeting ACVR2A and ACVR1B. Considering that the conservation of the miR-145-5p binding site of mammalian ACVR2A and ACVR1B and the homology of more than 98% between human and mouse of the two genes, the effects of T-MSC exosomes will be effective in human cells as well. Because activin A signaling initiated by co-activation of type II and type I, dual effects of miR-145-5p on those receptors may elicit synergistic effects to blunt activin A activity. Our in vivo data show significant upregulation of activin A receptors in activin A increased Bu-Cy mice. These results indicate that diverse pathologic conditions such as cancer cachexia, chemotherapy, and aging could increase activin A-mediated muscle atrophy through an increase in activin A and activin A receptors. Therefore, the targeting of activin A receptors may be a promising strategy to prevent or improve loss of skeletal muscle mass. Furthermore, activin A has positive effects on other type of differentiation, for example, follicular helper T cells [35] or neuronal differentiation of cortical neural progenitor cells [36]. Therefore, when trying to inhibit activin A receptors using T-MSC exosomes, the possible negative effects should also be considered in order to increase the therapeutic effects in accordance with their purpose. Although administration of bone marrow-derived MSCs have been shown to reduce muscle atrophy in high fat-diet mice [37], the effect of MSCs in protecting muscle mass has not been actively elucidated. Furthermore, the regulatory function of MSCs on activin A receptors is not known yet. Thus, it would be useful to check whether MSCs other than T-MSCs also have a blocking effect on activin A signals to expand availability of MSCs. The expression of miR-145-5p could be a correlate indicator, as WJ-MSCs showed significantly lower levels rather than T-MSCs in our study.

In summary, our data provide evidence that T-MSC exosomes influence the maintenance of skeletal muscle mass by targeting activin A receptors via miR-145-5p activity. These effects may be utilized by other muscle-affected various conditions in which activin A is increased.

## Figures and Tables

**Table 1:** Primers used for RT-PCR and qRT-PCR.

Primers	Sequences	Product Size (bp)
MyoD	F: 5′- CCACTCCGGGACATAGACTTG -3′ R: 5′- AAAAGCGCAGGTCTGGTGAG -3′	109
Myh1	F: 5′- GCGAATCGAGGCTCAGAACAA -3′ R: 5′- GTAGTTCCGCCTTCGGTCTTG -3′	138
Acvr2a	F: 5′- ATAAACGGCGACATTGTTTTGC -3′ R: 5′- TCGGTGTAACAGGATTTGAAGTG -3′	234
Acvr1b	F: 5′- CTGCCTACAGACCAACTACACC -3′ R: 5′- GCAGAAGTCAATATAGCAGCAGT -3′	190
Murf1	F: 5′- GTGTGAGGTGCCTACTTGCTC -3′ R: 5′- TGAGAGATGATCGTCTGCACT -3′	162
Atrogin1	F: 5′- CAGCTTCGTGAGCGACCTC -3′ R: 5′- GGCAGTCGAGAAGTCCAGTC -3′	244
Gapdh	F: 5′- GGTAAAGTGGATATTGTTGCCATCAATG -3′ R: 5′- GGAGGGATCTCGCTCCTGGAAGATGGTG -3′	173

## Data Availability

The datasets used and/or analyzed during the current study are available from the corresponding author on reasonable request.
